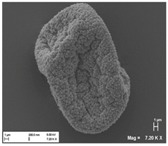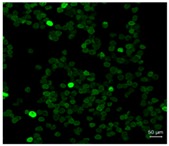# Combining MucilAir™ and Vitrocell^®^ Powder Chamber for the In Vitro Evaluation of Nasal Ointments in the Context of Aerosolized Pollen

**DOI:** 10.3390/pharmaceutics10020056

**Published:** 2018-05-10

**Authors:** Julia Metz, Katharina Knoth, Henrik Groß, Claus-Michael Lehr, Carolin Stäbler, Udo Bock, Marius Hittinger

**Affiliations:** 1 Department of Drug Delivery, PharmBioTec GmbH, 66123 Saarbrücken, Germany; j.metz@pharmbiotec.de (J.M.); k.knoth@pharmbiotec.de (K.K.); h.gross@pharmbiotec.de (H.G.); Claus-Michael.Lehr@helmholtz-hzi.de (C.-M.L.); m.hittinger@pharmbiotec.de (M.H.); 2 Department of Biopharmaceutics and Pharmaceutical Technology, Department of Pharmacy, Saarland University, 66123 Saarbrücken, Germany; 3 Helmholtz Institute for Pharmaceutical Research Saarland (HIPS), Helmholtz Center for Infection Research (HZI), 66123 Saarbrücken, Germany; 4 Department of Scientific Affairs Consumer Health, Bayer Vital GmbH, 51368 Leverkusen, Germany; carolin.staebler@bayer.com; 5 Bock Project Management, 54456 Tawern, Germany

**Keywords:** allergy prevention, pollen, aerosol deposition in vitro system, nasal mucosa, Bepanthen^®^ Eye and Nose Ointment

## Abstract

Hay fever is notoriously triggered when nasal mucosa is exposed to allergenic pollen. One possibility to overcome this pollen exposure may be the application of an ointment with physical protective effects. In this context, we have investigated Bepanthen^®^ Eye and Nose Ointment and the ointment basis petrolatum as reference while using contemporary in vitro techniques. Pollen from false ragweed (*Iva xanthiifolia*) was used as an allergy-causing model deposited as aerosol using the Vitrocell^®^ Powder Chamber (VPC) on Transwell^®^ inserts, while being coated with either Bepanthen^®^ Eye and Nose Ointment and petrolatum. No pollen penetration into ointments was observed upon confocal scanning laser microscopy during an incubation period of 2 h at 37 °C. The cellular response was further investigated by integrating the MucilAir™ cell system in the VPC and by applying pollen to Bepanthen^®^ Eye and Nose Ointment covered cell cultures. For comparison, MucilAir™ were stimulated by lipopolysaccharides (LPS). No increased cytokine release of IL-6, TNF-α, or IL-8 was found after 4 h of pollen exposure, which demonstrates the safety of such ointments. Since nasal ointments act as a physical barrier against pollen, such preparations might support the prevention and management of hay fever.

## 1. Introduction

Pollen allergy is a major public health problem worldwide and around 400 million people suffer from allergic rhinitis, according to the World Health Organization (WHO) [1]. The epidemiological research program “International Study of Asthma and Allergies in Childhood (ISAAC)” announced that the reasons for the worldwide spread of allergy cases are due to economic development, dietary and climate factors, infections, and pollen [2]. In addition to asthma and atopic eczema, allergic rhinoconjunctivitis (AR) as resulting from pollen as major allergens decreases the life quality of affected persons significantly [3,4]. The primary symptoms of AR that is caused by hay fever are sneezing, itching, secretion, and obstruction, followed by secondary and tertiary symptoms e.g., dyspnea, nasal hyperactivity up to conjunctivitis, sinusitis, and asthma [4].

Oral H1 antihistamines and intranasal corticosteroids build the two main classes of drugs for AR-treatment. A well-known and frequent property of oral treatment with H1 antihistamines is the negative impact on the nervous system, such as sedative effects [5]. These adverse effects were reduced in the second generation of H1 antihistamines, such as Cetirizine [6]. However, headaches, fatigue, and gastrointestinal distress were still observed [7]. Consequently, the demand for complementary and alternative medicines (CAM) for reducing allergy symptoms with less adverse effects is growing [8]. The research of new therapy strategies that are based on CAM is highly cost-intensive [9]. These CAMs are associated with a delayed response, which is not suitable in the acute case for reducing allergy symptoms. In addition to traditional CAMs against hay fever, such as homeopathic remedies [10] and nasal ointments, which act as a physical barrier to pollen and are able to provide a supportive effect without adverse effects that are similar to that of H1 antihistamines [11,12].

Bepanthen^®^ Eye and Nose Ointment has the indication to support wound-healing around the nose and eye area. The aim of this study was to investigate the potential of such ointments as CAM to prevent AR reactions by acting as a sole physical barrier to pollen, and thus preventing interaction with immune cells and subsequent allergy symptoms [13]. For this purpose, two innovative in vitro techniques were combined in this study: The Vitrocell^®^ Powder Chamber [14] and the human cell based MucilAir™ system [15,16,17]. Their combined use allows for the controlled application of aerosolized pollen on a human mucosal tissue surrogate, aiming to reflect a similar scenario in humans. In the first step, different pollens were selected with focus on their autofluorescence and their penetration depth into nasal ointments at the air-liquid interface. Second, the release of pro-inflammatory cytokines (IL-6, IL-8, and TNF-α) was quantified as an initial cellular response to pollen contact and to confirm the protective effect.

## 2. Materials and Methods

### 2.1. Microscopy

Size and shape of four selected pollen species, *Iva xanthiifolia*, *Populus nigra italic*, *Populus tremuloides*, and *Populus deltoids* (all from: Sigma-Aldrich, St. Louis, MO, USA) were examined through an electron microscope, the EVO HD 15 (Zeiss, Jena, Germany). Samples were sputtered with a thin gold layer using a Q150R ES sputter coater (Quorum technologies, Houston, TX, USA) before imaging at 5 kV and 7.5 mm working distance. A LSM 710 Axio Observer (Zeiss, Jena, Germany) visualized autofluorescence at a wavelength of 488 nm using an EC-Plan-Neofluar 10×/0.30M27 objective (Zeiss, Jena, Germany). ZEN 2 blue edition (Zeiss, Jena, Germany) was used as computer software. Additional wavelengths (405 & 561 nm) were applied when examining the ointment samples. For SEM imaging, MucilAir™ cell inserts were dehydrated with an ethanol (VWR Radnor, PA, USA) series at room temperature (RT) (70-80-90-96 (two times) and 100% for 10 min each), followed by sputter coating.

### 2.2. Parameters for Pollen Deposition with the Vitrocell^®^ Powder Chamber

**Figure 1:**
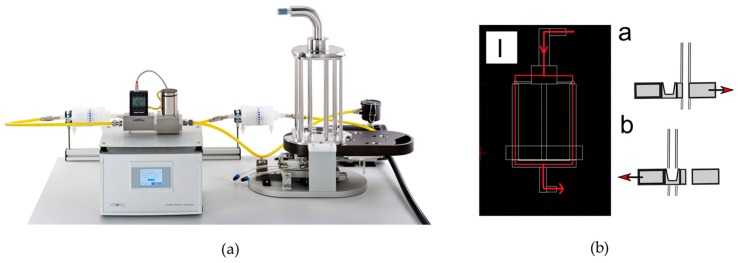
Vitrocell^®^ Powder Chamber (VPC) as an aerosol exposure system for pollen deposition. (**a**) composition of the VPC consisting of a tube for particle dosing, sedimentation tubes, exposure tray, and a controller [18]; (**b**) the air/pollen flow is indicated by the red line through the sedimentation tubes (I); the VPC can be switched from a sedimentation-mode (**a**) to an exposition mode; (**b**). The sedimentation mode (**a**) allows for passing of large particles before the cell system is switched under the particle filled tube (**b**). As pollen are comparable to large particles, the time in sedimentation mode was set to zero seconds. The system immediately switched to exposition mode and a maximum dose of pollen was deposited on the cells.modified from [14].

The Powder Chamber (VITROCELL^®^ Systems GmbH, Waldkirch, Germany) consists of four different parts (a particle release, sedimentation tubes, exposure tray, and a controller), as shown in Figure 1a. An aliquot of 20 mg pollen was aerosolized per run. The amount of pollen that was deposited could be controlled by varying the sedimentation tube length and the time in exposition and sedimentation mode, respectively (Figure 1b). The optimal parameters for pollen deposition in the VPC were: 30 L/min flow rate; a tube length of 30 cm, a sedimentation time of 0 sec and an exposition time of 15 min.

### 2.3. Penetration of Pollen into an Ointment Layer

**Figure 2:**
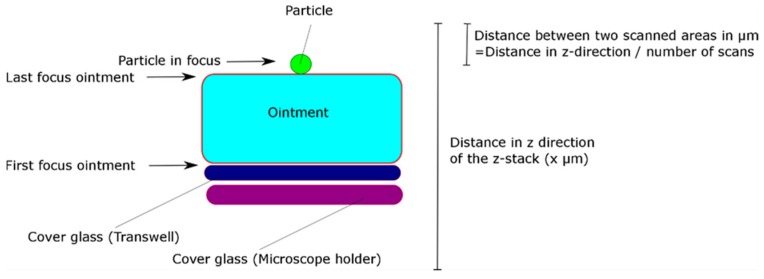
Situation for observing pollen penetration into ointment film preparation by confocal laser microscopy (CLSM) (not to scale).

The penetration depth of pollen into the Bepanthen^®^ Eye and Nose Ointment and petrolatum (both Bayer Vital GmbH, Leverkusen, Germany) served as an endpoint for evaluating the physical protective function of the ointments against pollen. Petrolatum is one of the main components in Bepanthen^®^ Eye and Nose Ointment. The penetration depth was determined by confocal laser microscopy (CLSM). Thus, the ointments were distributed in modified Transwell^®^ inserts (Corning, New York, NY, USA) where the membrane was replaced by a cover glass of similar size. A droplet (approx. 100 µL) of ointment was placed on the insert and was carefully distributed with a small brush. Transwell^®^ inserts used for comparison, were prepared in the same way, but without ointment. Three of the four deposition wells of the VPC were used as follows: (i) petrolatum, (ii) Bepanthen^®^ Eye and Nose Ointment, and (iii) control (empty insert). Pollen was aerosolized and deposited on the inserts. This experiment was conducted at room temperature and repeated three times, using fresh inserts for each cycle. Pollen penetration depth was measured by CLSM, as shown in Figure 2, within 30 min after the deposition experiments, keeping the samples on ice for transport. To follow pollen penetration over time, the samples were kept at 37 °C on a hot stage and CLSM z-stacks were taken at randomly chosen positions immediately (0 h, 1 h, and 2 h later).

### 2.4. Cytokine Release after Deposition on MucilAir™

**Figure 3:**
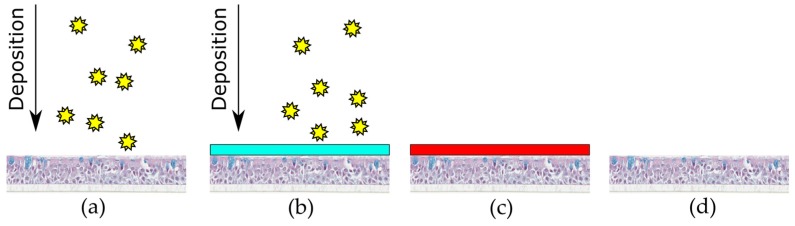
Experimental design for pollen deposition on MucilAir™. (**a**) pollen (symbolized by stars) deposited via VPC to MucilAir™ (n = 6); (**b**) pollen deposited via VPC and protected by Bepanthen^®^ Eye and Nose Ointment (light blue bar) to MucilAir™ (n = 6); (**c**) positive controls LPS stimulation on MucilAir™ (n = 3); and, (**d**) untreated negative control MucilAir™ in VPC (n = 3).

MucilAir™ cell cultures (Epithelix Sàrl, Genèva, Switzerland) were cultivated, according to manufacturer instructions, using the recommended medium under physiological air-liquid interface conditions (ALI). MucilAir™ is a reconstituted cell model based on human primary cells, which is known for its tight barrier, cytokine release, and mucus production [16]. After arrival, cells were cultured at the air-liquid interface for one day before the experiments were performed (at the air-liquid interface). 20 mg pollen with highest fluorescence intensity (*Iva xanthiifolia*) were deposited as aerosol on MucilAir™ with the aforementioned instrument settings. Three sham depositions without pollen (n = 1 MucilAir™ insert per cycle) were performed as negative control. Three MucilAir™ inserts were incubated with 10 μL lipopolysaccharides (LPS) (10 μg/mL, *Escherichia coli*, Sigma-Aldrich, St. Louis, MO, USA) in 990 µL 1 × DPBS (Gibco™, Thermo Fisher Scientific, Waltham, MA, USA) in the apical side (positive control). Six cycles were performed with one insert MucilAir™ protected by Bepanthen^®^ Eye and Nose Ointment and one insert without ointment protection in each cycle. In summary, three positive (LPS incubated samples) and three negative (sham deposition) controls, six wells that were protected by ointment and six wells without protection from deposited pollen were investigated (Figure 3). An inverse light microscope Primovert (Zeiss, Jena, Germany) visualized the distribution of pollen after deposition with the VPC on the MucilAir™ system. Transwell^®^ inserts were transferred into the VPC wells. As positive control 10 µg/mL LPS in 1 × DPBS (Waltham, MA, USA) was used to stimulate an inflammatory response in the MucilAir™ system [19,20]. Bepanthen^®^ Eye and Nose Ointment was pre-heated to 37 °C and was carefully added from the tube in a small droplet on top of the cell culture insert. The ointment was distributed carefully by using a sterile brush.

Figure 3 shows an experimental overview. Cytokines were quantified by Enzyme-linked Immunosorbent Assays (ELISA Kits, Thermo Fisher Scientific, Waltham, MA, USA). The release of the cytokines TNF-α, Interleukin-6, and Interleukin-8 was measured after pollen deposition on MucilAir™ and in both control groups 0, 4, and 24 h (sample volume: 200 μL). The ELISA kits were used according to the manufacturers’ guidelines.

## 3. Results

### 3.1. Pollen Selection

Table 1 lists the investigated pollen with focus on their size, shape (SEM) and characteristic autofluorescence (CLSM). All pollen had a size of approximately 30 μm and a similar behavior within the Powder Chamber was expected, accordingly. *Iva xanthiifolia* was selected for further experiments because of its strong autofluorescence and its known allergic potential and increasing relevance [15].

### 3.2. Penetration of Pollen into an Ointment Layer

Pollen from the *Iva xanthiifolia* was deposited with the VPC and the deposited amount of pollen was counted on modified, untreated Transwell^®^ inserts. 31 ± 19 pollen were detected per CLSM image corresponding to 4231 ± 2592 pollen/cm^2^. The results indicate that the pollen deposition process using the VPC has certain variability depending on the position and inter-cycle differences. The ointment thickness (petrolatum) was measured by CLSM and had a range of 20–250 µm. The z-focus of the instrument of the CLSM Bepanthen^®^ Eye and Nose Ointment was limited to approximately 100 µm penetration depth (signal was scattered by excipients).

**Figure 4:**
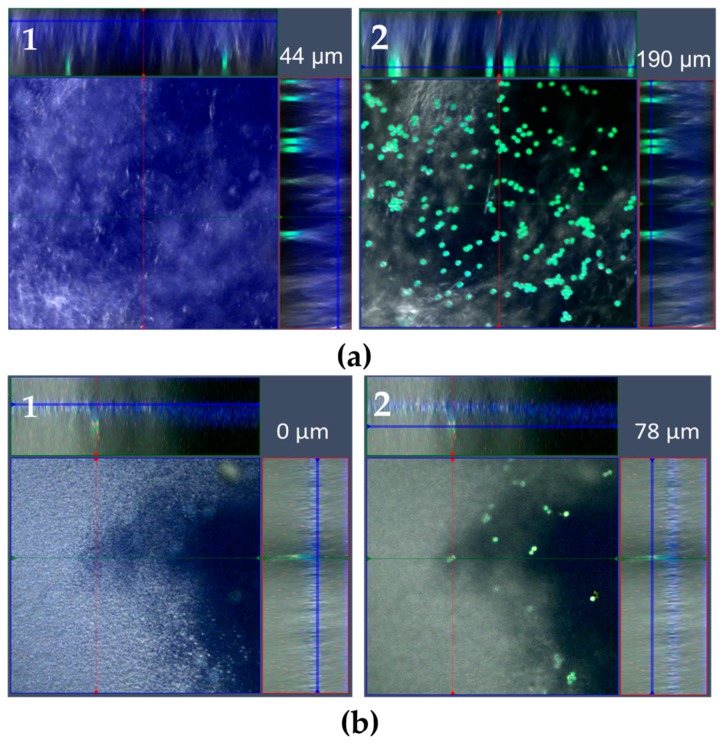
Pollen deposition of the *Iva xanthiifolia* (z-stack by CLSM). (**a**) *Iva xanthiifolia* deposited via VPC on petrolatum. Image 1 shows the focus area of the ointment beginning with a high of 44 µm by performing the z-stack. Image 2 on the right shows the focus area of the pollen (green), which is the last area in which ointment is localized (190 µm). Consequently, no penetration of pollen was observed. The blue scattered light from the laser further stresses the thickness of the ointment. (**b**) Bepanthen^®^ Eye and Nose Ointment via CLSM. Image 1 indicates the first focus level of the Bepanthen^®^ Eye and Nose Ointment on the cover slip, image 2 demonstrates the second focus level on the top of the Bepanthen^®^ Eye and the Nose Ointment with deposited pollen. The images were selected from a z-stack, which emphasizes the limited fluorescence signal of pollen in the ointment. The ointment (beginning left, 0 µm) and pollen on top (right, 78 µm) highlight both limiting imaging points.

The penetration depth of *Iva xanthiifolia* into the Bepanthen^®^ Eye and Nose Ointment and petrolatum was examined over two h at 37 °C after pollen deposition. No significant penetration of pollen was observed into either the Bepanthen^®^ Eye and Nose Ointment or the petrolatum, demonstrating that both ointments act as a physical barrier to pollen, which is shown in Figure 4: The first focal plane, on which the first autofluorescence signal appeared, was designated as the start of the ointment layer (Figure 4a,b, figures left (1), ointment in blue). The last autofluorescence signal from the ointment was detected on the same level when the autofluorescence signal of pollen was measured (Figure 4a,b, right (2), pollen in green), which emphasize that no penetration occurred. The calculated distance is similar to the ointment thickness applied.

### 3.3. Cytokine Release after Deposition on MucilAir™

**Figure 5:**
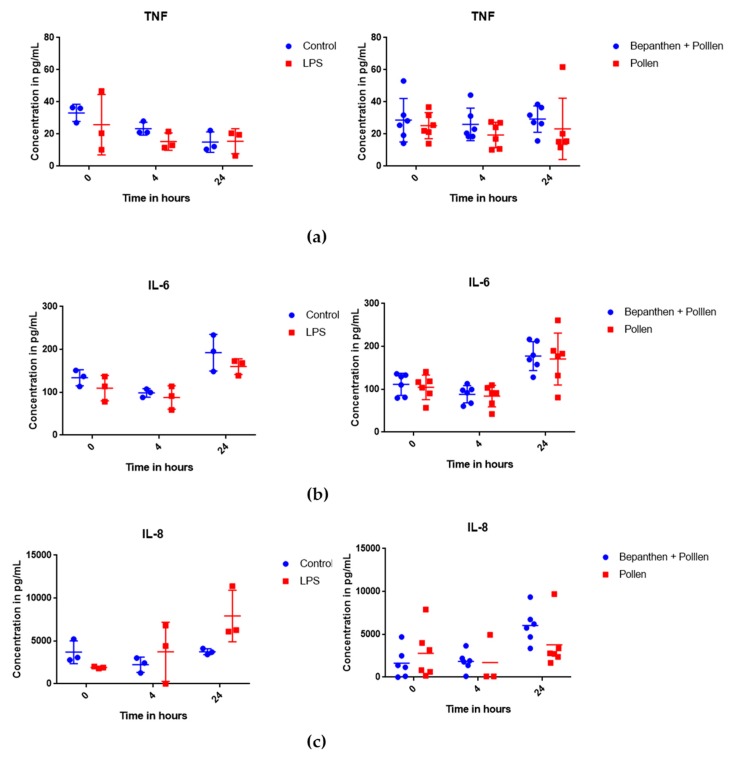
Cytokine release of MucilAir™ with pollen deposition on Bepanthen^®^ Eye and Nose Ointment (n = 6 per time point and group) and control with lipopolysaccharides (LPS) stimulation over 24 h (n = 3 per time point and group); (**a**) release of TNF-α; (**b**) release of Interleukin-6; and, (**c**) release of Interleukin-8 (dilution of 1:1000).

The deposition of pollen on the epithelial layer was proven by light microscopy. Some visible cilia beating and a slow movement of pollen was observed. Most of the pollen were located at the edges after 24 h when the last cytokine samples were taken. Figure 5 summarizes the levels of TNF-α, IL-6, and IL-8. The first diagram shows the control group with LPS stimulation against a negative control group and the second diagram shows the results from the treatment with Bepanthen^®^ Eye and Nose Ointment against the negative control group. No differences were found for IL-6 and TNF-α for LPS stimulation, as well as for treatment with Bepanthen^®^ Eye and Nose Ointment (Figure 5a,b). The IL-8 release was increased after LPS stimulation as compared to the control group without stimulation. Bepanthen^®^ Eye and Nose Ointment has no influence on the IL-8 release over 24 h (Figure 5c). The detected concentrations of IL-6 and TNF-α were low and did not allow for further analysis. After 24 h, a significant increase of IL-8 was observed for the LPS group when compared to the control group, which implicates a moderate reaction of the MucilAir™ system. The treatment of Bepanthen^®^ Eye and Nose Ointment did not influence the release of cytokines, nor did it trigger an inflammatory response of MucilAir™.

**Figure 6:**
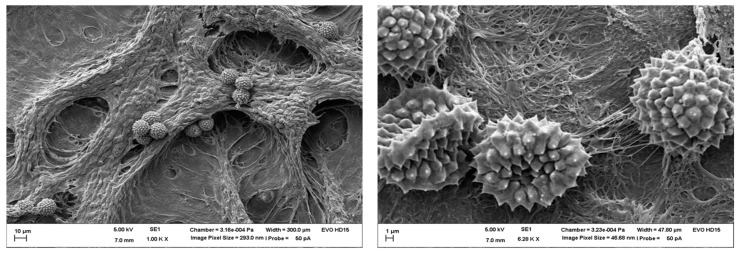
Scanning electron microscopy (SEM) images after deposition with VPC of pollen from *Iva xanthiifolia* on MucilAir™. Left: SEM picture of *Iva xanthiifolia* on MucilAir™ with an image pixel size of 293.0 nm and a magnification of 1.000×; right: SEM picture *Iva xanthiifolia* on MucilAir™ with an image pixel size of 46.68 nm and a higher magnification of 6.280×.

SEM was performed to visualize pollen with the resulting cell culture interactions (Figure 6). The images indicated pollen, which sticks to mucus and cellular like structures.

## 4. Discussion

The aim of this study was to investigate the protective effects of nasal ointments acting as a physical barrier against aerosolized pollen. We decided for the combination of an innovative aerosol deposition system (VPC) in combination with the MucilAir™ cell culture model of human nasal mucosa. Due to its prominent autofluorescence and due to its allergic potential and the future importance in EU [21], *Iva xanthiifolia* was selected among other pollen species that are listed in Table 1. A different behavior as aerosol in the Powder Chamber was not expected as all investigated pollen were of similar size. The VPC allowed for an efficient and homogenous aerosolization, and the deposition of this pollen. However, no penetration of pollen could be observed in either petrolatum or Bepanthen^®^ Eye and Nose Ointment when spread on glass slides. This finding already suggests some kind of protective effect of such nasal ointments. In such settings however, any physiological clearance due to mucociliary beating and mucus are missing [24].

Various in vitro models simulating the mechanical and the functional facets of the upper respiratory system in vivo are available. These models can be divided into cell culture models, like primary cells or nasal tissue cells, excised nasal tissues, and different experimental set-ups regarding the permeation pathways [24]. The overall goal of these models is to come as close as possible to the in vivo conditions failing on the reconstitution of the complex communication and the interaction of the different components of nasal mucosa. Hence, the MucilAir™ cell culture systems were developed to set-up for such experimental designs by consisting of differentiated and functional cells of the human airway epithelium with goblet cells, which produce mucus, basal cells, and ciliated cells [16,19,25]. In our study, the VPC was combined for the first time with MucilAir™ model to address this biological complexity. The observed ciliated movement of pollen to the edges of the Transwell^®^ membrane within 24 h combined with SEM images of mucus-trapped pollen emphasized a functional mucociliary clearance (MCC) within the in vitro system. In vivo, the MCC, and the mucus layer itself are for nasal drug delivery one of the limiting factors resulting in impeded drug permeability [11,24]. Mucus is of utmost importance, since the ciliated cells transport the mucus to nasopharynx in order to remove foreign particles [26]. Bepanthen^®^ Eye and Nose Ointment influences the physiological function of the mucociliary system of the upper respiratory tract in our study that is caused by building an additional layer on the system. In this in vitro setup, the ointment has the same function as the physical barrier to tag the pollen by passing the nose by air. One of the main side action of Bepanthen^®^ Eye and Nose Ointment is that the cilia’s function of transporting pollen might be decreased. Nevertheless, this should be a minor factor that is caused by the early positioning of the layer in the very first contact area of the nose. The effect on the morphological change or possible disruption of the physiological nasal mucosa by the application of the substance should be considered in further studies.

The deposition of allergenic pollen lead to symptoms of allergy and inflammation processes in the immune system [13,27], resulting in an increased pro-inflammatory cytokine release. The MucilAir™ system is suitable for the investigation of potential allergic cytokines, as it has already been used as an examination system for respiratory sensitizers [15]. According to our hypothesis, the application of Bepanthen^®^ Eye and Nose Ointment should decrease the release of typical involved cytokines, such as IL-6, TNF-α, and IL-8. However, the release of IL-6 and TNF-α was not influenced by any of the chosen conditions. This might be due to a comparatively low number of immune cells present in the currently available in vitro models of the nasal mucosa. It has been shown, that the MucilAir™ releases IL-8 after stimulation with LPS [15]. Consequently, LPS was applied as a pro-inflammatory mediator to the MucilAir™ [19,28], leading to an increased IL-8 secretion after 24 h. Interestingly, the mean of IL-8 secretion was even higher after the treatment with Bepanthen^®^ Eye and Nose Ointment and deposited pollen when compared to the control group. This might be caused by associated mechanical stress on the cellular layer itself. Pollen did not stimulate any cytokine release (IL-8, TNF-α and IL-6), which is based on a protective mucus layer, as indicated by the SEM images. Furthermore, the concentration of 10 µg/mL LPS might be further increased to stimulate the MucilAir™ system in order to generate a more pronounced response [29]. Nonetheless, in an inflammatory state the properties of mucus change and the native barrier might be less efficient, which is hard to mimic in vitro [30,31,32].

This study combines two innovative in vitro systems for respiratory research, the MucilAir™ and the VPC to address the in vivo situation of pollen deposition. The set-up offered air-liquid experimental conditions, including differentiated and functional cells of the human airway epithelium with goblet cells, basal cells, and ciliated cells [16,19,25]. However, further adjustments, such as the implementation of other cell types e.g., immunological cells are necessary to address interactions with pollen in vitro and to enhance the readout.

## 5. Conclusions

This study was designed to prove the capacity of nasal ointments in vitro to act as a physical barrier against pollen, and subsequently reduce allergic potential. The combination of the in vitro model MucilAir™ with the air-liquid deposition instrument VPC for the application of dry particles (pollen) was a first step in the direction of a relevant in vitro test method for nasal ointment efficacy studies. We demonstrated that nasal ointments are able to physically prevent the penetration of pollen. Conversely, we were neither able to confirm nor negate any reduction of the allergenic potential of pollen by the application of nasal ointments in terms of cytokine release profiles by using the MucilAir™ system with IL-6, IL-8, and TNF-α as read-out markers making further adjustments necessary. The combination of the VPC with MucilAir™ can nevertheless be seen as a promising step on the way to more complex models addressing the air-liquid interface.

## Figures and Tables

**Table 1:** Overview of the characteristics, size, shape, and autofluorescence of used pollen.

Pollen Species	Characteristics	Scanning Electron Microscopy	Confocal Light Scanning Microscopy
*Iva xanthiifolia*	Size: ~28 µm Shape: serrated, rough surface Allergic potential: allergenic Neophyt [21]	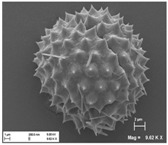	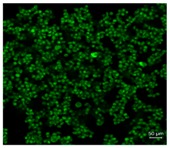
*Populus nigra italic*	Size: ~26 µm Shape: uneven, particulate surface Allergic potential: ♂ dioecious [22]	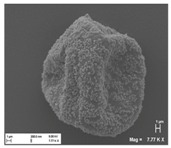	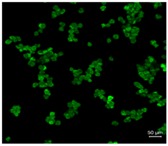
*Populus tremuloides*	Size: ~30 µm Shape: ovate, particulate surface Allergic potential: not known	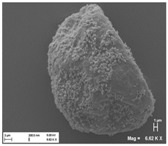	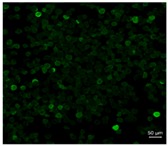
*Populus deltoides*	Size: ~28 µm Shape: longish, porous surface Allergic potential: yes [23]